# Affected Ovary Relative Volume: A Novel Sonographic Predictor of Ovarian Reserve in Patients with Unilateral Endometrioma—A Pilot Study

**DOI:** 10.3390/jcm9124076

**Published:** 2020-12-17

**Authors:** Stefano Cosma, Andrea Roberto Carosso, Martina Moretto, Fulvio Borella, Domenico Ferraioli, Marialuisa Bovetti, Fiammetta Gervasoni, Claudia Filippini, Alberto Revelli, Simone Ferrero, Chiara Benedetto

**Affiliations:** 1 Gynecology and Obstetrics 1, Department of Surgical Sciences, City of Health and Science, University of Turin, 10126 Turin, Italy; stefano.cosma@unito.it (S.C.); andrea88.carosso@gmail.com (A.R.C.); alberto.revelli@unito.it (A.R.); chiara.benedetto@unito.it (C.B.); 2 Department of Surgical Sciences, City of Health and Science, University of Turin, 10126 Turin, Italy; more.marti90@gmail.com (M.M.); fulvio.borella87@gmail.com (F.B.); marialuisa.bovetti@gmail.com (M.B.); fiammetta.gervasoni@gmail.com (F.G.); 3 Department of Oncology Surgery, Léon Bérard Comprehensive Cancer Center, 69008 Lyon, France; ferraioli.domenico@gmail.com (D.F.); claudia.filippini@unito.it (C.F.); 4 Academic Unit of Obstetrics and Gynaecology, IRCCS Ospedale Policlinico San Martino, 16132 Genova, Italy

**Keywords:** antral follicle count, antimüllerian hormone, endometrioma, endometriosis, fertility, premature ovarian failure, ovarian reserve, ultrasonography

## Abstract

Background: The assessment of ovarian reserve in the case of endometrioma is of pivotal importance for planning a tailored management. However, both the antral follicle count (AFC) and the antimüllerian hormone (AMH) dosage are subject to a fair degree of variability in ovarian endometriosis. This study aimed to identify a sonographic parameter of ovarian reserve that could implement current available markers in patients with unilateral endometrioma. Methods: Patients with unilateral endometrioma admitted to our Endometriosis Center between March 2018 and April 2019 were enrolled. Transvaginal ultrasonography for the evaluation of eight sonographic indicators and AMH level determination were performed. The relationship between AMH level and each indicator was assessed. Results: Thirty-four women were included. There was a positive significant correlation between AMH level and the healthy ovary AFC (HO-AFC) (*r* = 0.36 *p* = 0.034). A stronger, negative correlation between AMH level and the ratio between the volume of the affected and the healthy ovary (affected ovary relative volume, AORV) (*r* = −0.47; *p* = 0.005) was evidenced. AORV had a satisfactory accuracy (AUC 0.73; CI 0.61–0.90; *p* = 0.0008), and the cut-off value of 5.96 had the best balance of sensitivity/specificity in distinguishing between patients with a good ovarian reserve (AMH ≥ 2 ng/mL) and those at risk of ovarian reserve depletion after excisional surgery. Conclusion: AORV may be a useful tool to assess ovarian reserve in patients with unilateral endometrioma without previous surgery and to guide physicians in clinical management.

## 1. Introduction

Endometriosis affects approximately 10% of the general female population [1]. About 40% of subfertile women with endometriosis are diagnosed with an ovarian endometriotic cyst or endometrioma [2,3], with controversial effects on spontaneous conception [4], as well as detrimental outcomes on oocyte quality [5,6] and assisted reproductive techniques (ART) [7,8,9].

Ovarian reserve can be evaluated with ultrasound and serological markers. Ultrasound markers provide the antral follicle count (AFC) of both ovaries and the ovary volume (OV). Serological markers include the measurement of basal follicle-stimulating hormone (FSH) and the antimüllerian hormone (AMH).

Ultrasound evaluation of ovarian reserve is challenging when an ovarian endometrioma is large because of its interposition, while serological AMH levels should not be influenced. However, conflicting evidence for the effect of endometrioma on AMH levels has been reported: A recent review showed much lower AMH levels in patients with endometrioma than in patients with benign lesions or healthy controls [10]. Differently, other reports found no correlation between endometrioma per se and ovarian reserve [11]. Finally, an increase in AMH levels related to the presence of ovarian endometrioma has been reported [12].

Owing to conflicting evidence, it is not clear whether AMH levels alone are a reliable indicator of ovarian reserve in women with endometrioma. Furthermore, AMH assay variability and the lack of a standardized international assay are daily concerns for reproductive medicine specialists [13]. The aim of the present study was to evaluate the diagnostic potential of a new sonographic parameter to implement current ovarian reserve markers in patients with unilateral endometrioma. We define this new marker as the affected ovary relative volume (AORV), i.e., the ratio between the volume of the affected and the healthy ovary. Also, we suggest a cut-off value of AORV that may be useful for identifying patients at risk of impaired ovarian reserve after excisional surgery and that may guide clinicians in the management of such patients.

## 2. Materials and Methods

Women with ovarian endometriosis referred to our Endometriosis Center, Department of Surgical Sciences, City of Health and Science, University of Turin, between March 2018 and April 2019, were enrolled in the study after providing written, informed consent. The exclusion criteria were bilateral endometrioma, prior ovarian surgery, the presence of other benign ovarian cysts or endometrioma with atypical sonographic features, ongoing hormonal contraceptive use, polycystic ovary syndrome (PCOS), and premature ovarian failure (POF) according to European Society for Human Reproduction and Embryology (ESHRE) guidelines [14,15].

Transvaginal ultrasonography and a blood test for AMH determination were performed on the same morning during the early follicular phase of a spontaneous cycle (i.e., between cycle days 2 and 5). Scans were performed with a 6–12 MHz transducer on a GE Voluson E8 System (GE Healthcare, Milwaukee, WI, USA) by a physician with extensive experience in pelvic ultrasound.

**Figure 1:**
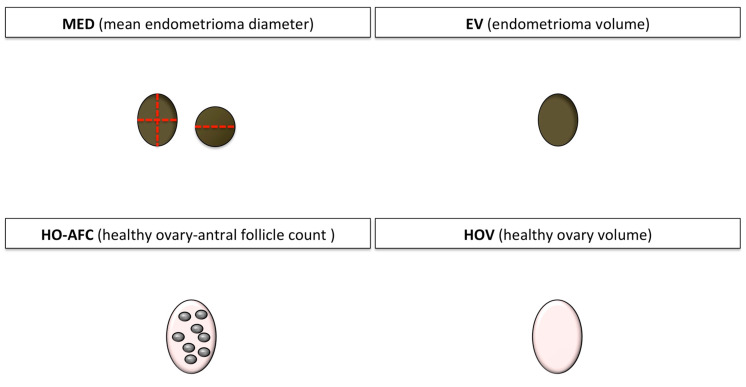
Schematic diagram of the four absolute sonographic indicators. Brown circle: Endometrioma; dotted red line: Measure line; grey circle: Antral follicle; pink oval: Contralateral healthy ovary.

**Figure 2:**
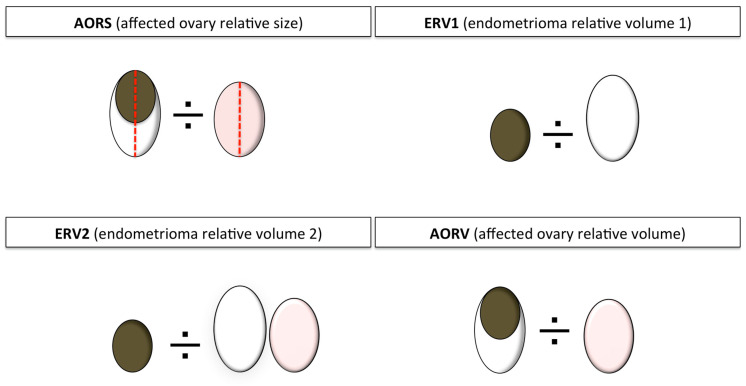
Schematic diagram of the four relative sonographic indicators. Brown circle: Endometrioma; dotted red line: Measure line; pink oval: Contralateral healthy ovary; white oval: Affected ovary.

The antral follicle count (AFC) of the participants was assessed. The size of the ovary and the endometrioma was defined by three diameters: Maximum diameter in the transverse, the anteroposterior, and the longitudinal axes. Four absolute and four relative sonographic indicators were obtained (Figure 1 and Figure 2):

- Mean endometrioma diameter (MED);
- Endometrioma volume (EV);
- Healthy ovary AFC (HO-AFC);
- Healthy ovary volume (HOV);
- Affected ovary relative size (AORS), i.e., the ratio between the largest diameter of the affected and the healthy ovary;
- Endometrioma relative volume 1 (ERV1), i.e., the ratio between the EV and the volume of the affected ovary;
- Endometrioma relative volume 2 (ERV2), i.e., the ratio between the EV and the volume of both ovaries; and
- Affected ovary relative volume (AORV), i.e., the ratio between the volume of the affected and the healthy ovary.

If multiple endometriomas were detected in the same ovary, the indicator was reported as the sum of the individual indicators of each endometrioma.

Serum AMH level was determined using a commercially available ELISA kit (AMH Gen II ELISA, Beckman Coulter, Brea, CA, USA) according to the manufacturer’s instructions. The AMH concentration in each sample was determined by interpolation from a standard curve and calculated as ng/mL as recommended. The reference range established by our laboratory was 0.1–6 ng/mL [16]. All exams were evaluated by the same physician. The AMH cut-off was 2 ng/mL as the threshold to identify women who can be safely referred to surgery (AMH ≥ 2) without the risk of significant depletion of ovarian reserve. This value was chosen because it is linked to the impact of surgery on ovarian reserve if enucleation of unilateral endometrioma is performed (30% decrease in AMH) [17] and because it is related to the definition of “poor responder” in patients undergoing in vitro fertilization (IVF) treatment (AMH values between 0.5 ng/mL and 1.3 ng/mL) [18,19]. Based on these figures, we obtained the value according to the equation [(x − 30%x) = 1.3]. The result (1.9) was rounded up to 2 ng/mL.

Quantitative variables were expressed as the mean ± standard deviation (SD) and qualitative categorical variables as frequency and percentages. Pearson’s *r* coefficient was run to assess the relationship between the ovarian reserve based on AMH level, and the sonographic variables (MED, EV, HO-AFC, HOV, AORS, ERV1, ERV2, AORV). Spearman’s correlation coefficient (ρ) was also used to measure the strength and direction of association between AORV ratio and AMH. Accuracy of parameters to discriminate between poor and good ovarian reserve was evaluated using receiver operating characteristic (ROC) curve analysis. The area under the ROC was calculated and a 95% confidence interval (CI) was given. Diagnostic thresholds for significant parameters were proposed on Youden’s index, which balanced maximum test sensitivity and test specificity. A *p*-value of 0.05 was set as statistically significant. Statistical analyses were performed using SAS software ver. 9.4 for Windows (SAS Institute, Carey, NC, USA).

The Institutional Review Board of the City of Health and Science of Turin approved the study (reference number 0040486/00152).

## 3. Results

**Figure 3:**
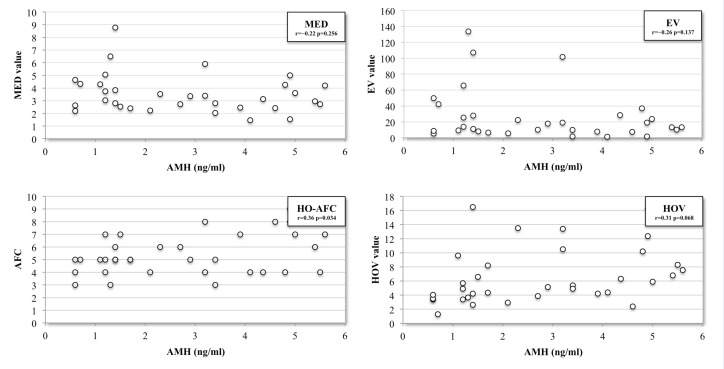
Scatterplots of the four absolute sonographic indicators. Pearson’s correlation coefficient (*r*) testing the strength of the association between the independent variable (antimüllerian hormone (AMH) level) and the dependent variables (indicators), ranging from −1 to +1. EV: endometrioma volume; HO-AFC: healthy ovary AFC; HOV: healthy ovary volume; mean endometrioma diameter (MED)

**Figure 4:**
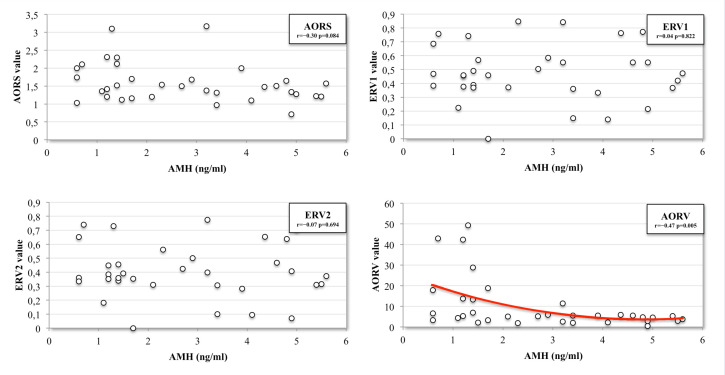
Scatterplots of the four relative sonographic indicators. Pearson’s correlation coefficient (*r*) testing the strength of the association between the independent variable (AMH level) and the dependent variables (indicators), ranging from −1 to +1. AORS: affected ovary relative size; AORV: affected ovary relative volume; ERV1: endometrioma relative volume 1; ERV2: endometrioma relative volume 2

Seventy-eight Caucasian patients with endometrioma were evaluated: 15/78 (19.2%) were diagnosed with bilateral endometriomas, 8 (10.2%) with PCOS, 2 (2.5%) with concomitant serosal cysts, and 5 (6.4%) were receiving hormonal therapy and were excluded from the study. The remaining 34 patients met the inclusion criteria. The mean age was 33.4 ± 3.8 years: 20.6% were <30 years old, 32.3% between 30 and 34 years, and 47.1% ≥35 years. The mean body-mass index (BMI) was 23.2 ± 4.3 kg/m^2^, and 6/34 (17.6%) were smokers. Six (8.1%) were diagnosed with more than one endometrioma per ovary. The mean blood AMH level was 2.76 ± 1.6 ng/mL, 3.43 ± 1.8 ng/mL in women less than 30 years old, 2.8 ± 1.9 ng/mL between 30 and 34 years old, and 2.41 ± 1.4 ng/mL in women aged 35 or over. The mean AFC of the affected and healthy ovary was 3.62 ± 1.7 and 5.53 ± 1.5, respectively. The mean size of the endometriomas was 3.5 ± 1.5 cm. A not significant relationship was found between AMH levels and the following volumetric indicators: EV (*r* = −0.26; *p* = 0.137), MED (*r* = −0.22; *p* = 0.256), LED (*r* = −0.22; *p* = 0.213), ERV2 (*r* = −0.07; *p* = 0.694), and ERV1 (*r* = 0.04; *p* = 0.822). A weak not significant correlation was found between blood AMH level and AORS (*r* = −0.36; *p* = 0.082) and HOV (*r* = 0.31; *p*= 0.068). There was a positive significant correlation between AMH level and HO-AFC (*r* = 0.36 *p* = 0.034), but a stronger, negative correlation between AMH level and AORV (*r* = −0.47; *p* = 0.005) was evidenced (Figure 3 and Figure 4). Indeed, the two variables have a significant nonlinear relationship, as suggested by the Spearman correlation coefficient (ρ = −0.48 *p* = 0.004), which was slightly higher than the Pearson’s (*r* = −0.46 *p* = 0.005).

**Figure 5:**
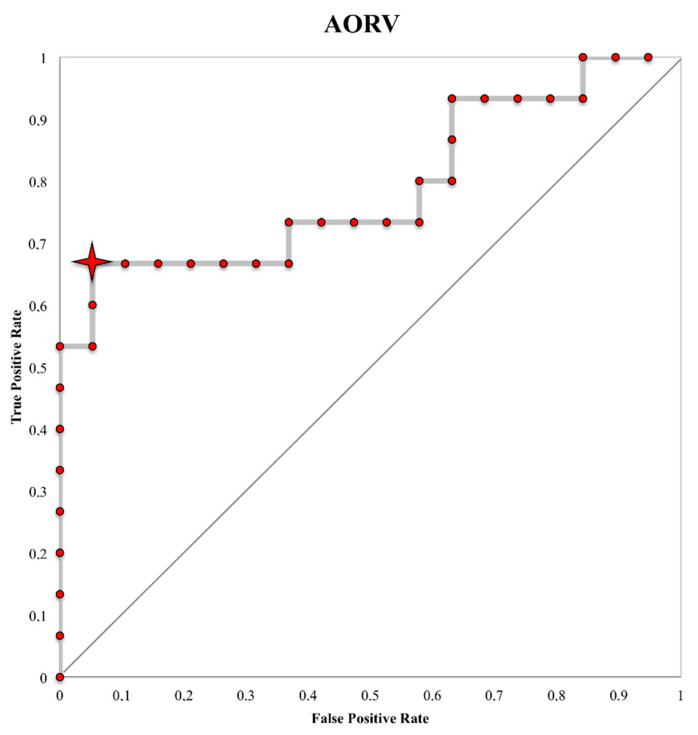
Affected ovary relative volume (AORV) receiver operating characteristic (ROC) curve at the antimüllerian hormone (AMH) cut-off of 2 ng/mL. Red star, AORV cut-off value of 5.96.

The area under the curve (AUC) for AORV was s 0.73 (CI 0.61–0.90; *p* = 0.0008). The diagnostic threshold in the ROC curve with the best balance of sensitivity/specificity was 5.96 for AORV according to the results obtained from the Youden index. An AORV >5.96 had a sensitivity of 66.7% and a specificity of 94.7% in detecting women at risk of significant diminished ovarian reserve after excisional surgery (AMH < 2 ng /mL) (Figure 5).

## 4. Discussion

The absence of a reliable correlation between the effects of ovarian endometrioma and ovarian reserve markers means that referral to surgery is usually based on a dimensional criterion (most frequently ≥ 3 cm) [20,21,22]. However, because the dimensional criterion alone is unable to integrate the patient’s reproductive potential, this aspect must be considered before proposing surgical treatment to a patient with endometriosis, regardless of whether she is planning pregnancy or not [23,24]. Indeed, women with a poor baseline ovarian reserve undergoing surgery on the basis of the dimensional criterion alone are exposed to the risk of surgical diminished ovarian reserve, in addition to endometriosis-related damage, resulting in severe impairment of ovarian reserve and potential infertility [25,26,27].

The identification of an innovative volumetric cut-off that could be used for tailoring clinical management could aid in this delicate decision, so long as it can be integrated with the estimated overall ovarian reserve of the candidate for surgery. Our study indicated the potential statistical significance of AORV, a volumetric predictor. If the AORV is <5.96, symptomatic patients with unilateral endometrioma have a good ovarian reserve and can be offered surgery without the fear of further compromising the ovarian reserve. Otherwise, if the AORV is >5.96, counseling and preservation methods should be recommended to the symptomatic patient, while the infertile woman with a desire for motherhood should be addressed to ART rather than surgery and/or to spontaneous attempt to conceive [28].

Our findings underscore the relevant importance of the healthy contralateral ovary, which constitutes a woman’s individual ovarian reserve and reflects the condition before the disease. Unlike intermenstrual volumetric asymmetric variations, high or low ovarian reserves are shared symmetrically between the two ovaries without altering the ratio. However, the value of the healthy ovary becomes relevant only in relation to the affected one. The weaker relationship between AMH and HOV or HO-AFC compared to AMH and AORV is probably justified by the fact that the assessment of the healthy ovary does not take into account the residual ovarian reserve of the affected ovary, which is closely conditioned by the endometrioma volume.

Factor other than simple mechanical tissue stretching by cysts might be responsible for reduced follicular density in the cortex surrounding endometriomas. The reactive inflammation with the increase in tissue oxidative stress may result in massive fibrosis and the loss of cortex-specific stroma that maintains follicular nests. A decline in the antral follicle count may be associated with focal loss of AMH, and consequently, with activation of primordial follicles that undergo atresia. The size of the cyst may correlate with the duration and severity of the inflammatory reaction in normal cortical tissue within endometriomas [29,30]. The relationship between the number of preantral follicles and the ovarian volume [31] and the histopathologic reason for their reduction as the cyst grows make AORV a reliable predictor of ovarian reserve in patients with unilateral endometrioma and provide a useful tool for its use with other indicators of ovarian reserve such as AMH and AFC.

Nonetheless, the limits of AMH and AFC need to be carefully considered. Serum AMH levels are proportional to the number of preantral and small antral follicles in the ovaries. Therefore, they are considered one of the most important indirect clinical markers of ovarian reserve [32,33]. In patients with ovarian endometriosis, the validity of AMH is questionable because the relative contribution of the affected and the intact ovaries cannot be easily discriminated. Furthermore, recent data have shown significant fluctuations of AMH in several conditions and questioned whether a single measurement is reliable for clinical decision-making. The AMH level also appears to vary with a variety of factors such as hormonal contraceptive use, pregnancy, body-mass index, and smoking status [34]. Analytical variations due to differences in sample storage conditions and/or assay methods could further complicate this scenario [35]. Finally, an intracycle variation of 20.7% in AMH over the natural ovulatory cycle and an intercycle variation of 28% have been reported according to the results of a fully automated AMH assay [36].

AFC by ultrasound is the only way of assessing reserve of each ovary for most cases, but its role in the presence of endometrioma is controversial. Some authors have suggested that AFC in ovaries with endometrioma is likely to be underestimated, since the number of oocytes retrieved is higher than expected based on the AFC. The underestimation of AFC is attributed to the impairment of the transvaginal probe resolution due to endometrioma [37]. A meta-analysis of two retrospective studies reported that women with in situ endometrioma had similar AFC compared to women without endometriosis [38]. In contrast, a prospective study reported that women with endometrioma had significantly lower AFC compared to healthy women with no endometriomas [26]. For this reason, among the indicators, we tested the AFC of the healthy ovary (HO-AFC), which was theoretically not impaired by the detrimental effects related to endometrioma’s presence.

A further limitation of these two assessment methods is their clinical interpretation. ART specialists prefer the clinical application of AMH and AFC. This is particularly true when we consider that the validation of these measures is mostly based on the number of oocytes retrieved after controlled ovarian stimulation, i.e., treatments that are carried out only in IVF-dedicated units. The “superspecialization” of modern gynecology, and of medicine as a whole, makes it difficult for an excellent surgeon to deal equally well with reproductive medicine and IVF. The result is that treatment is often managed according to “personal clinical experience” rather than based on standardized measurements that can integrate complex decision algorithms that would require a multispecialist approach. The easy execution and the identification of a “reliable” cut-off for identifying patients at higher risk of diminished ovarian reserve also make AORV an affordable and straightforward tool for nonreproduction specialists unfamiliar with AMH and AFC. Furthermore, AORV could be assessed in every phase of menstrual cycle, while AFC should be performed only in the early follicular phase.

As previously said, many authors believe ovarian endometriosis could technically affect the counting of follicles, leading to underestimation of AFC, thus burdening this tool with false-positive patients [37,39]. Using a cut-off of 5.96, our sonographic predictor can identify a woman with AMH levels < 2 in 94.7% of cases, while it identifies women with an acceptable AMH before surgery (i.e., ≥2 ng/mL) in 66.7% of cases. The probability of a poor ovarian reserve with a positive test value is 83.3%, while the likelihood of a good ovarian reserve with a negative result is 81.8%.

Our study has several limitations. The sample size was relatively small due to the restrictive eligibility criteria. Furthermore, it is unclear whether AORV can be used in patients undergoing hormone therapy. Although the hormonal dependent-volumetric reduction is shared symmetrically between the two ovaries, the affected ovary is potentially less involved because the endometrioma is less sensitive to medical treatment, suggesting that the AORV volumetric ratio in patients undergoing hormonal therapy might be overestimated.

The test is not useful for women with PCOS. Serum AMH is higher in women with PCOS than in healthy women [40], reflecting the increased number of small antral follicles in which AMH production is highest. In these patients, the ovarian volume is very large such that small cysts do not cause significant volume changes. Undoubtedly, the main limitation of our test is that it cannot be applied in cases of bilateral lesions.

The strengths of our study are the rigorous inclusion criteria, standardized AMH measurement during the first days of the follicular phase (previously defined as the best “window” to predict ovarian response to controlled stimulation) [36,41], and the same laboratory assay performed by the same physician, thus limiting interpersonal variability in AMH dosage.

Further research might give the volumetric ratio (AORV), a dynamic value that expresses the variation of the ovarian reserve, together with the change of the index. Women with endometrioma experience a dramatic, progressive decline in serum AMH levels, which is faster than that in healthy women. The decrease in serum AMH level rate is reported to be more than 20% after 6 months in patients with unilateral endometrioma [42]. For example, the distance from the cut-off could be interpreted as a measure of the time still available to attempt spontaneous conception before the cyst grows further and depletes the ovarian reserve.

In conclusion, this preliminary study shows that AORV is a reliable means by which to assess ovarian reserve in patients with unilateral endometrioma without previous surgery and to guide physicians in clinical management. Ongoing validation will include the application of this quick tool in daily outpatient care.
